# Metabolic Diffusion in Neuropathologies: The Relevance of Brain-Liver Axis

**DOI:** 10.3389/fphys.2022.864263

**Published:** 2022-05-12

**Authors:** Sergio Vegas-Suárez, Jorge Simón, María Luz Martínez-Chantar, Rosario Moratalla

**Affiliations:** ^1^ Department of Pharmacology, Faculty of Medicine and Nursing, University of the Basque Country (UPV/EHU), Leioa, Spain; ^2^ Cajal Institute, Spanish National Research Council (CSIC), Madrid, Spain; ^3^ Network Center for Biomedical Research in Neurodegenerative Diseases (CIBERned), Carlos III Institute of Health (ISCIII), Madrid, Spain; ^4^ Liver Disease Lab, Center for Cooperative Research in Biosciences (CIC BioGUNE), Basque Research and Technology Alliance (BRTA), Derio, Spain; ^5^ Centro de Investigación Biomédica en Red de Enfermedades Hepáticas y Digestivas (CIBERehd), Carlos III Institute of Health (ISCIII), Madrid, Spain

**Keywords:** brain, liver, neuroinflammation, ketone bodies, Parkinson’s Disease and Alzheimer’s Disease, ammonium

## Abstract

Chronic liver diseases include a broad group of hepatic disorders from different etiologies and with varying degrees of progression and severity. Among them, non-alcoholic fatty (NAFLD) and alcoholic (ALD) liver diseases are the most frequent forms of expression, caused by either metabolic alterations or chronic alcohol consumption. The liver is the main regulator of energy homeostasis and metabolism of potentially toxic compounds in the organism, thus hepatic disorders often promote the release of harmful substances. In this context, there is an existing interconnection between liver and brain, with the well-named brain-liver axis, in which liver pathologies lead to the promotion of neurodegenerative disorders. Alzheimer’s (AD) and Parkinson’s (PD) diseases are the most relevant neurological disorders worldwide. The present work highlights the relevance of the liver-related promotion of these disorders. Liver-related hyperammonemia has been related to the promotion of perturbations in nervous systems, whereas the production of ketone bodies under certain conditions may protect from developing them. The capacity of the liver of amyloid-*β* (A*β*) clearance is reduced under liver pathologies, contributing to the development of AD. These perturbations are even aggravated by the pro-inflammatory state that often accompanies liver diseases, leading to the named neuroinflammation. The current nourishment habits, named as Western diet (WD) and alterations in the bile acid (BA) profile, whose homeostasis is controlled by the liver, have been also related to both AD and PD, whereas the supplementation with certain compounds, has been demonstrated to alleviate the pathologies.

## Graphical Abstract

The role of the liver in the metabolism of toxic compounds and in A*β* and α-synuclein clearance is related to neurological disorders such as Alzheimer’s Ddisease (AD) and Parkinson’s Disease (PD) development, although the supplementation with certain compounds may exert a protective effect. The current Western diet and alterations in the BA profile, whose homeostasis is controlled by the liver, have been also related to both AD and PD.
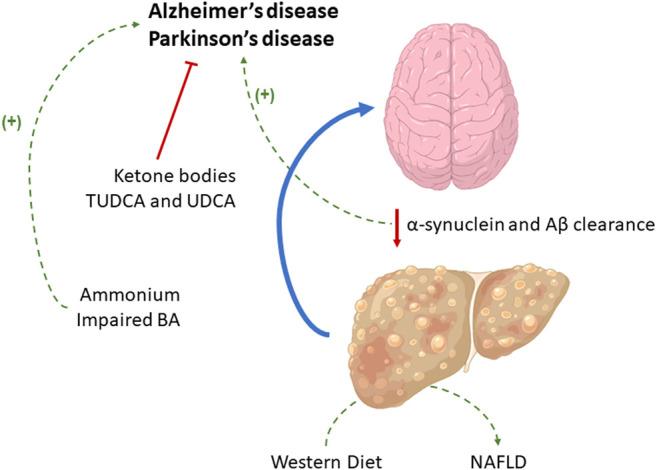

## 1 Liver Diseases

Chronic liver diseases include a broad group of hepatic disorders from different etiology, in which a characteristic progression leads to severe stages such as cirrhosis or liver cancer. With the appearance of Sovaldi for treating hepatitis C (HCV) and the subsequent reduction of its incidence (Lam et al., 2014), non-alcoholic fatty liver disease (NAFLD) and alcoholic liver disease (ALD) have become the main cause of chronic liver pathologies.

NAFLD, which is newly noted as metabolic-associated fatty liver disease (MAFLD) due to its metabolic etiology, has become a major health problem in the world. Younossi and others who have estimated its prevalence to be 20–30% worldwide, expect a steeper increase within next years due to current lifestyle habits and western diet (WD) (Younossi et al., 2018). NAFLD/MAFLD consists of a clinical syndrome that comprises a group of disorders characterized by a slow progression, where the disruption of lipid homeostasis causes a benign condition named steatosis or fatty liver (NAFL). NAFL development can be followed by second hits that contribute to the aggravation of the pathology: Oxidative stress by reactive oxygen species (ROS), endoplasmic reticulum stress, mitochondrial dysfunction, and lipid toxicity. NAFL aggravation may lead to non-alcoholic steatohepatitis (NASH), characterized by cell death, inflammation, and fibrosis development (Simon et al., 2020b). Cirrhosis and an increased risk of developing liver cancer occur at later stages of the spectrum of pathologies associated with NAFLD/MAFLD (Gao and Tsukamoto, 2016). Altogether, cirrhosis and liver cancer represent around 3.4% of overall deaths, with 1.7 patients affected worldwide (Saviano and Baumert, 2019).

Additionally, the exposure to toxic compounds such as drugs or alcohol can induce hepatic pathologies. In this context, ALD has become the leading cause of liver disease in many countries such as the United States where alcoholism affects around 61% of its population with a 10–12% people diagnosed as heavy drinkers (Hussen et al., 2018). Chronic alcohol consumption of 30–50 g/day causes ALD, with steatosis in 90% patients who drink more than 60 g/day and cirrhosis in 30% of cases (Mathurin and Bataller, 2015). The exceeded hepatic capacity of metabolizing xenobiotics and the subsequent exhaustion of antioxidant mechanisms in the hepatocyte leads to the development of the pathology. This pathology is commonly characterized by a mitochondrial dysfunction, glutathione depletion and hepatocyte death (Ramachandran et al., 2018).

Altered hepatic homeostasis during liver pathologies may promote either the release of several compounds or their accumulation in the organism (Romero-Gómez et al., 2015). The systemic circulation can make those compounds reach other organs leading to perturbations. Considering the existing liver-brain connection, several neurological disorders have been widely linked to liver pathologies, in which their presence is often associated (Beraza & Trautwein, 2008). (Poulose et al., 2017; Wahl et al., 2019; Popa-Wagner et al., 2020).

## 2 Neurodegenerative Disorders

Apart from liver diseases, pathologies that affect neural system present one of the biggest challenges for public health and current medicine. They account for an elevated worldwide incidence and prevalence that, like liver pathologies, grow rapidly due to the long-life expectancy. Neurodegenerative disorders are also characterized by a progressive, irreversible, and chronic degeneration of the central and peripheral nervous systems (CNS and PNS, respectively) (Villemagne et al., 2013; Kalia & Lang, 2015). The World Health Organization (WHO) has estimated that by 2050 more than 130 million people will be affected by neurodegenerative illnesses, with Alzheimer’s disease (AD) and Parkinson’s Disease (PD) as the two most prevalent disorders (Nichols et al., 2019). Although aging is considered the main risk factor for neurodegenerative disorders, environmental factor and lifestyle have also an important influence on the risk of late-onset forms of the diseases (Wahl et al., 2019; Popa-Wagner et al., 2020).

AD affects approximately 40 million people worldwide and it is expected to be doubled by 2030. AD is the sixth highest cause of mortality in the United States, and it is clinically characterized by the progressive cognitive decline, impaired learning ability and memory function, as well as mood disturbances (Brodaty et al., 2011; Cheng et al., 2020). Although the sporadic form is the most prevalent, AD has a strong genetic component and several research lines have been focused on identifying genetic causes and risk factors in AD. Polymorphism in the apolipoprotein E gene seem to be the major genetic risk factor for the late-onset AD. However, rare early-onset autosomal dominant forms are also known (König and Stögmann, 2021). This pathology is considered as multifactorial, where pathophysiological brain changes are related to the accumulation of misfolded proteins. Particularly, the characteristic neuronal loss involves two different molecular mechanisms: the extracellular insoluble senile plaques mainly composed by amyloid-*β* peptide (A*β*) and intraneuronal neurofibrillary tangles formed by phosphorylated Tau (Gulisano et al., 2018). Although vascular factors play a role, ROS production and neuroinflammation are considered as primary contributors to AD pathogenesis, some evidence points to the liver as a possible origin of A*β* deposits observed in the disease (Bassendine et al., 2020; Knopman et al., 2021). Pathological A*β* accumulation observed in AD may be due to an impaired hepatic A*β* degradation due to impaired balance featuring accumulation of amyloid fibrils of *α*-synuclein (Lam et al., 2021; Maarouf et al., 2018) suggesting that liver may play a key role in the onset and progression of AD (Section 4). Conversely, viral infection with Human Herpesvirus 1/2, Cytomegalovirus or HCV have also been associated with dementia and AD in patients who are genetically susceptible (Balin & Hudson, 2018; Bassendine et al., 2020). Otherwise, epidemiological studies have demonstrated that other pathologies such as metabolic syndrome (MetS) or type 2 diabetes (T2DM) are also important risk factors for AD development in aging (Stoeckel et al., 2016; Bello-Chavolla et al., 2019).

The second most common neuropathy is PD, with a global prevalence estimated around 2% of the worldwide population over 60 years and rising to 3% in those over 80 (Tysnes & Storstein, 2017). Due to the increasing life expectancy, PD prevalence and incidence are growing and, like AD, expected to be doubled by 2030 (Lee & Gilbert, 2016). The most diagnosed cases represent idiopathic or sporadic PD, while familiar or genetic forms are less frequent, accounting for less than 15% of total cases (Balestrino & Schapira, 2020). The gradual dopaminergic degeneration of *substantia nigra pars compacta* has been considered to be the main hallmark of PD (Kish et al., 1988). However, the presence of Lewy bodies composed by *α*-synuclein may also damage other brain regions in early stages of the disease in both patients with PD and animal models (Braak and Tredici, 2017; Vegas-Suarez et al., 2019; Gómez-Benito et al., 2020; Paredes-Rodriguez et al., 2020). Adding to this, ROS production, metabolic alterations and impaired mitochondrial activity also play a relevant role during PD pathogenesis and the vulnerability of dopaminergic neurons (Puspita et al., 2017; Zhang et al., 2018). These processes can be stronger in PD cases caused by genetic mutations and, glucocerebrosidase (GBA1) mutations represent the most common genetic risk factor for PD (Garcia-Sanz et al., 2017; Garcia-Sanz et al., 2021). Diabetes and PD may share some mechanism. Thus, increased striatal oxidative stress and altered dopamine neurotransmission leading to nigrostriatal neurodegeneration were observed in both diabetic and parkinsonian mice (Pérez-Taboada et al., 2020). Like AD, infections with either HCV or other agents such as influenza or pneumonia have also been related with the onset of the disease as triggers of chronic microglial inflammation (Alam et al., 2016; Cocoros et al., 2021). Viral infections (Beatman et al., 2015), alterations in gut microbiota often linked with liver diseases (Bajaj et al., 2012; Keshavarzian et al., 2020) or neuroinflammation and intestinal inflammation, can induce *α*-synuclein overproduction with the subsequent microglial over-reactivity.

Considering the link between liver and brain, the present review aims to highlight the main contributions of this organ to the development of neuropathies. Moreover, considering the effect of diet-induced metabolic alterations over chronic liver pathologies, the relationship between neurological disorders and food intake is also reviewed in this work.

## 3 Hepatic Released Compounds and Neurological Disorders

Although the liver maintains nutrient and energy homeostasis in the organism, the development of liver pathologies is often accompanied by the release of certain compounds that lead to associated morbidities. Herein, several metabolites participate in the development of neurogenerative disorders.

One of the most characterized hepatic compounds is ammonium, whose excess is produced as an imbalanced homeostasis by either: An increased glutaminase (GLS)-mediated production through glutamine degradation (Romero-Gómez et al., 2009; Simon et al., 2020a) or reduced scavenging by decreased glutamine synthetase (GLUL) expression (Soria et al., 2019) or urea cycle activity (De Chiara et al., 2018). In the brain, astrocytes are the main contributors of glutamine synthesis by removing the cation from the media, thus leading to a cascade of neurochemical events that often cause hepatic encephalopathy (HE) (Butterworth, 2013). Moreover, ammonia accumulation generates free radicals that promote post-translational modifications, such as nitrotyrosination or nitrosylation, on certain brain proteins (Oja et al., 2017). The first one, nitrotyrosination, often leads to a loss-of-function or the inhibition of tyrosine phosphorylation (Picón-Pagès et al., 2019), whereas nitrosylation consists of the binding of a nitric oxide (NO) molecule that modulates the catalytic activity of the enzyme (Kumar et al., 2010). Herein, cyclins are the most frequent protein modified thus inducing cell death (Kumar et al., 2010).

Microglial and astroglioma cells have also been characterized to promote phagocytosis and endocytosis under ammonia accumulation, with subsequent modification in cytokine secretion and an increased lysosomal hydrolases activity (Atanassov et al., 1995). The release of hepatic pro-inflammatory cytokines also promotes the development of neuropathologies such as HE in patients with cirrhosis (Seyan et al., 2010). In this context, neuroinflammation is a common feature that accompanies liver failure, which leads to the reactivity of microglia and increases the synthesis of other pro-inflammatory cytokines, promotes the recruitment of monocytes, and even alters the blood-brain barrier (BBB) permeability (Butterworth, 2013). The existing interconnection between liver and gut has also been characterized by many researchers such as Bajaj and others, who have correlated poor cognition and endotoxemia in liver disease patients who had previously developed HE (Bajaj et al., 2012).

Under calorie restriction conditions there is an enhanced *β*-oxidation that promotes the synthesis of ketone bodies. Otherwise, these compounds are also highly produced in ketogenic diets consisting of a macronutrient distribution of 55–60% fat, moderate proteins, and very low carbohydrates (Roehl et al., 2019). Interestingly, ketone bodies have been reported to have an effect over the brain metabolism and function in the development of neurodegenerative disorders (Jensen et al., 2020). Ketone bodies can enter the BBB through the action of monocarboxylate transporters (Pierre and Pellerin, 2005) to be converted into acetyl-CoA in neuronal and glial cells. Remarkably, a study from Henderson and co-workers has demonstrated in a clinical study that the supplementation with the ketogenic compound AC-1202 prevented AD development in a cohort of 152 (Henderson et al., 2009). Related to this, ketone bodies might have beneficial effects by improving mitochondrial efficiency in neuronal cells, supplementing the normal glucose reliance of the organ (Henderson, 2008). Moreover, additional studies point out their relevance in preventing AD as well, by either promoting hepatic ketogenesis or supplementing the patients with these metabolites (Thickbroom, 2021). Particularly, the ketone bodies-mediated inhibition of histone deacetylases (HDAC) may promote an enhanced antioxidant production while improving the mitochondrial oxidative phosphorylation and ROS reduction (Yang et al., 2019). Herein, Huang and others have characterized that the ketone body *β*-hydroxybutyrate exerts if effect over HDAC in microglial cells (Huang et al., 2018). Otherwise, ketone bodies can be produced not only in the liver, but also in astrocytes as several studies highlight. This fact may imply that astrocytes promote ketogenesis in a cytoprotective way (Guzmán & Blázquez, 2001) whereas microglial-mediated effect of ketone bodies may also protect from neuropathologies.

## 4 Disrupted Amyloid Beta (A*β*) Metabolism During Hepatopathologies

The liver plays a key role in the removal of toxic compounds and exogenous antigens; therefore, liver pathologies affect the development of neurological disorders such as AD or PD. Specific virus display neurotropic effects particularly affecting the *substantia nigra* and promoting the aggregation of *α*-synuclein (Bosanko et al., 2003). The development of liver pathologies also provokes depletion in the hepatic immune response that aggravates the phenotype exerted by viral infections.

Regarding the clearance of A*β* from the system, there are three main pathways that mediate the efflux of this molecule from the brain to periphery and accounts for approximately about 50% of total brain clearance (Bassendine et al., 2020). The brain uptake of circulating A*β* is mediated by specific receptors in brain endothelium (Shibata et al., 2000), while lipoprotein receptor protein-1 (LRP-1) seizes around 70–90% of circulating A*β* in the liver (Sagare et al., 2007). The reduced A*β* deposits detoxification during liver pathology increased the circulating levels of the compound, leading to accumulation in the brain and promoting the appearance of plaques and derived symptoms (Estrada et al., 2019). Functional LRP-1 is located in hepatic endothelial cells from the sinusoid, and it contributes to the rapid removal of its blood ligands, showing specificity for A*β*. However, added to the reduced capacity in A*β* clearance, alterations lead to a breakdown of the BBB function as it also regulates the tight junction proteins in the endothelial cells in the brain barrier (Zhao et al., 2016).

As aforementioned, the hepatic capacity in eliminating toxic substances is decreased in liver pathologies causing the aggravation of neurological disorders such as AD (Estrada et al., 2019). Under a hepatic pro-inflammatory state frequently observed in liver pathologies, the reduced LRP-1-mediated A*β* uptake is correlated with increased serum IL-6 levels and circulating A*β* (Wang et al., 2017). AD development is inversely correlated to the peripheral metabolism of A*β* (Lam et al., 2021) but directly related to the amount of circulating plasma lipoproteins, with triglyceride-rich in particular. Indeed, the possibility of formatting triglyceride-rich lipoprotein-A*β* complexes and their extravasation may lead to cerebral capillary amyloid angiopathies (Matsubara et al., 2004). Thus, the number of circulating triglycerides and cholesterol is correlated with A*β* levels rather than other liver markers such as transaminases (Bosoi et al., 2021). Amyloidosis is also accelerated in atherogenic diets in an animal model of pre-symptomatic AD consisting of amyloid precursor protein/presenilin 1 (APP/PS1) mice. The parenchymal retention of triglyceride-rich lipoprotein-A*β* complexes promote a pro-inflammatory phenotype that exacerbates AD (Ettcheto et al., 2016). Supporting this concept, PD could start to be considered as a systemic amyloidosis featuring accumulation of amyloid fibrils of *α*-synuclein rather than localized amyloidosis as it might happen in AD (Araki et al., 2019). Cholesterol may be also involved in the pathogenesis of PD. Thus, previous studies from our laboratory have shown that N370S GBA1 mutation alters the lysosomal enzymatic activity leading the accumulation of glucosylceramide and of cholesterol which are related to the expression of multilamellar bodies in fibroblasts derived from patients with PD (García-Sanz et al., 2017; García-Sanz et al., 2021).

Regarding AD, Bossio and others recently demonstrated that a high-fat diet (HFD) modulates hepatic A*β* and cerebrosterol metabolism using a triple transgenic mouse model of AD (Bosoi et al., 2021). Cerebrosterol is the principal way of eliminating brain cholesterol and once produced in brain, the liver is the main organ responsible for cerebrosterol glucuronidation or sulfation by eliminating it through bile acids and urine (Björkhem, 2006). The shift that occurs during HFD from lipogenesis towards glucose production leads to a disruption of the metabolism of both A*β* and brain cholesterol (Tang et al., 2016).

## 5 Western Diets: Liver Metabolism and Neurodegenerative Disorders

The study from Bosoi highlights the liver as the main organ responsible for systemic metabolic homeostasis. Therefore, and considering the relationship between liver and neurologic pathologies, it is expected that dietary habits have an impact over both type of diseases. Neural circuits that are involved in feeding pattern show a precise coordination with brain centers that modulate the energy homeostasis and the cognitive function (Gómez-Pinilla, 2008). The ingestion of food triggers the release of peptide hormones such as insulin or glucagon-like peptide 1 (GLP-1) (McNay, 2007) that regulate system metabolism. Particularly, GLP-1 reaches the hypothalamus and hippocampus in order to activate signal-transduction pathways that promote synaptic activities and contribute to learning and associative and spatial memories (During et al., 2003). Nourishment habits and physical activity have also an impact over the brain-derived neurotrophic factor (BDNF) and insulin-growth factor-1 (IGF-1) production. BDNF is related to metabolism and synaptic plasticity, whereas IGF-1 supports nerve growth differentiation, synaptic plasticity and neurotransmitter synthesis and release. The development of hyperglycemia and insulin resistance, which often have a metabolic origin due to bad dietary habits, leads to a reduction of IGF-1 production and an impairment of cognitive function (Anlar et al., 1999; Gómez-Pinilla, 2008; Torres Aleman, 2012; Herrero-Labrador et al., 2020).

The development of liver pathologies impairs hepatic A*β* clearance so that liver diseases, especially chronic ones, are often linked to neuropathologies. Particularly NAFLD is associated with a lower cognitive performance and affecting several types of memory (Celikbilek et al., 2018), where the metabolic perturbations that occur imply an imbalance between lipid-clearing and –increasing mechanisms. Herein, fatty acid composition has reported to have an effect over cerebral functionality of plasma-derived apolipoprotein B (ApoB)-containing lipoproteins (Takechi et al., 2010). Moreover, nutritional changes have been widely reported to have an impact over NAFLD development (Perdomo et al., 2019). Macronutrient composition of a diet associated with NAFLD often show common features: Higher saturated fatty acids (SFA) and lower polyunsaturated fatty acids (PUFA), fiber and vitamins C and E (Musso et al., 2003). Among PUFA, omega-6-rich diets promote NAFLD development (Cortez-Pinto et al., 2006) whereas omega-3, together with other nutrients such as vitamins C and K or folates, inversely correlates to NAFLD (Han et al., 2014). It is evident that an excessive calorie intake promotes obesity development and a subsequent increased risk for NAFLD, while glucose or other simple sugars consumption also promote hepatic lipogenesis causing steatosis and triggering NAFLD (Wehmeyer et al., 2016).

Furthermore, current WD often avoid the recommended nutrients to prevent hepatopathologies: MUFAs, omega-3 PUFAs, vegetable proteins, pre- and pro-biotics, resveratrol, coffee, taurine, and choline (Perdomo et al., 2019). Current WD may be the main contributor of the increasing incidence of NAFLD, which has been additionally pointed out as another possible trigger of AD (Wiȩckowska-Gacek et al., 2021a; Więckowska-Gacek et al., 2021b). Although WD-associated nourishment pattern does not have a unified list of the components, current different WDs share a SFA content around 35–60% and elevated amounts of simple sugars such as sucrose or fructose. Ultra-processed food, refined ingredients and simple carbohydrates are other common features of this diet. The existing gut-liver-brain axis is also affected by WD, as the gut functionality is altered by reducing the correct absorption of some required nutrients and vitamins (McMillin et al., 2015). Related to the brain and nervous system, hippocampal functionality can be perturbed by the fat and sugar present in WD. Hippocampal-dependent learning and memory are reduced with a high-SFA and sugar-containing diet in both rodents (Abbott et al., 2019) and patients (Attuquayefio et al., 2017) which are characterized by a reduction in long-term potentiation (Jena et al., 2018). Additionally, the size of hippocampus has been reported to be decreased under WD in 60–64 old men, showing a higher amount of phosphorylated Tau proteins associated to neurofibrillary pathologies such as AD (Xu et al., 2017). WD also accelerates the appearance of A*β* (Wiȩckowska-Gacek et al., 2021a), while synaptic plasticity may be also reduced by the WD-associated reduction in acetylcholine, dopamine, gephryin, serotonin, synaptophysin, or BDNF (Jena et al., 2018).

Moreover, WD also induces a lipid accumulation in adipocytes that meanwhile induces a pro-inflammatory state that further aggravates neuropathologies. There is an increased secretion and activity of peripheral pro-inflammatory cytokines by adipocytes (Ali et al., 2020), while gut dysbiosis of gut microbiome triggered by WD contributes to further enhancing the inflammation (Dabke et al., 2019). Related to this, the composition of circulating free fatty acids is of relevance as they can activate pro-inflammatory toll-like receptors TLR-2 and TLR-4 to activate inflammation pathways and promote the secretion of cytokines: tumor necrosis factor (TNF), interleukin 1*β* and 6 (IL-1*β* and IL-6) or macrophage chemoattractant protein-1 (MCP-1) among others (Więckowska-Gacek et al., 2021b).

## 6 Bile Acids in Brain Function

Bile acids (BA) are mainly synthesized in the liver in two pathways by the different cytochrome P450 isoforms (CYP) and using cholesterol as substrate. The classic pathway implies the action of CYP7A1 which catalyzes cholesterol hydroxylation into 7-*α*-hydroxycholesterol, being further hydroxylated by: CYP8B1 producing cholic acid (CA) as product or CYP27A1 producing chenodeoxycholic acid (CDCA) as final product. The other pathway involves CYP27A1 and CYP7B1 for CDCA formation. Furthermore, there is an additional extra-hepatic neural pathway that converts brain cholesterol by the action of CYP46A1 and CYP39A1 (Lorbek et al., 2012).

During WD there are dysregulated BA homeostasis and dysbiosis that contribute to systemic inflammation, microglial reactivity, and reduced neuroplasticity. In the previously cited work from Jena and others in 2018 a reduction in *Bdnf* mRNA expression and postsynaptic density protein 95 (PSD-95), markers of brain functionality, were found reduced in both brain and microglia (Jena et al., 2018). BA also take part in the clearance of circulating A*β*, whose uptake is realized by the LRP-1 and the low-density lipoprotein receptor (LDLR) that are highly expressed in hepatocytes (Kanekiyo and Bu, 2014). However, perturbations in BA have an effect over neuropathologies as lithocholic acid (LCA) has been detected in brain during an experimental model of multiple sclerosis (Naqvi et al., 1969), whereas elevations in of serum BA have been linked to neuropathological states such as HE. They have been also found in brain tissue and cerebrospinal fluid (Bron et al., 1977). Other bile acids (CA, DCA, and CDCA) modulate neurotransmitters such as N-methyl D-aspartate (NMDA) or *γ*-amino-butyric acid (GABA), acting through ligand-gated ion channels for inhibiting their action (Schubring et al., 2012). CA, CDCA and LCA decrease respiratory timing while DCA influences respiratory patterns (C. Zhao et al., 2014). Under elevated BA there is a inhibition of hepatic glucocorticoid clearance and this causes a significant disruption in the hypothalamic-pituitary-adrenal (HPA) axis (McNeilly et al., 2010). The opening of the BBB leads BAs to enter the brain *via* the bile acid transporter apical-sodium dependent bile acid transporter (ABST) and, related to this, TCA and CDCA have been reported to activate the glucocorticoid receptor (GR) in neurons (McMillin and DeMorrow, 2016). In the context of BA metabolism, the signaling of foresaid X-receptor (FXR) is essential, as its deletion impairs memory and motor coordination leading to changes in GABA; glutamate, norepinephrine and serotonin neurotransmission (F. Huang et al., 2015).

BA have been widely linked to neurological disorders such as AD or PD. In the context of AD, an altered profile has been found in mild cognitive impairments that happen prior to AD (Nho et al., 2019). Surprisingly, the supplementation with certain compounds has appeared to alleviate the pathology. The administration of tauroursodeoxycholic acid (TUDCA) and ursodeoxycholic acid (UDCA) have been reported to suppress A*β*-induced apoptosis in neurons by modulating the E2F-1/P53/BAX pathway (Rodrigues et al., 2000). The determination of glycoursodeoxycholic acid (GUDCA) could be of interest for the prediction of the onset of AD or amnesic mild cognitive impairment (Mapstone et al., 2014). Similarly, the metabolomics profiling of BA also seems to be a potential biomarker for PD (Graham et al., 2018). Metabolic alterations have been found in plasma of patients with familiar and idiopathic PD (Yakhine-Diop et al., 2020), where under a BA disruption microbiota converts them into toxic derived compound that promote PD pathogenesis (Li et al., 2021). The supplementation of UDCA reduces the development of the disease in preclinical models of PD (Mortiboys et al., 2015). Finally, BA have been also linked to other neurological disorders such as HE, cerebrotendinous xanthomatosis, traumatic brain injury, stroke, and amyotrophic lateral sclerosis (McMillin & DeMorrow, 2016).

## 7 Conclusion

This work evidences the involvement of liver diseases in the development and progression of neurodegenerative disorders. The release of toxic compounds by the liver aggravates both AD and PD development, whereas ketogenesis may lead to their prevention. The role that the liver has in the metabolism of toxic compounds is of relevance in A*β* clearance, as liver dysfunction promotes the circulating levels of this compound. The maintenance of the BBB integrity is also crucial for protecting the nervous system against degeneration, as impairments in the barrier are related to the presence of certain compounds in the brain. In this context, brain BA are related to neurological disorders although the supplementation with certain compounds has been demonstrated to exert a protective effect. Finally, the nourishment habits are of relevance as they may alter liver physiology thus promoting the appearance of pathologies such as NAFLD, contributing to neurological disorders.
